# Infarct Zone Circumferential Strain Independently Predicts Left Ventricular Functional Recovery After ST-Segment Elevation Myocardial Infarction: A Multiparametric CMR Study

**DOI:** 10.3390/diagnostics16131992

**Published:** 2026-06-26

**Authors:** Agneta Virbickiene, Olivija Dobiliene, Arnoldas Leleika, Justina Jureviciute, Paulius Bucius, Neda Jonaitiene, Egle Kazakauskaite, Lina Bardauskiene, Vacis Tatarunas, Tomas Lapinskas

**Affiliations:** 1 Institute of Cardiology, Lithuanian University of Health Sciences, LT-50161 Kaunas, Lithuania; vacis.tatarunas@lsmu.lt; 2 Heart Center, Medical Academy, Lithuanian University of Health Sciences, LT-50161 Kaunas, Lithuania; olivija.dobiliene@lsmu.lt (O.D.); paulius.bucius@lsmu.lt (P.B.); lina.bardauskiene@lsmu.lt (L.B.); tomas.lapinskas@lsmu.lt (T.L.)

**Keywords:** ST-segment elevation myocardial infarction, cardiac magnetic resonance, circumferential strain, feature tracking, infarct size, left ventricular remodeling, T1 mapping, prognosis

## Abstract

**Background/Objectives**: Cardiac magnetic resonance (CMR) imaging provides a comprehensive assessment of myocardial injury after ST-segment elevation myocardial infarction (STEMI), yet the relative prognostic value of segmental infarct zone parameters compared with global indices for predicting left ventricular (LV) functional recovery remains incompletely defined. This study aimed to determine whether segmental infarct zone functional and structural CMR parameters provide prognostic information for LV functional recovery after a first STEMI treated with primary percutaneous coronary intervention (PCI). **Methods:** In this prospective single-center study, 93 patients with a first STEMI (median age 61 years; 77% male) underwent CMR at baseline (median 3 days post-PCI) and at a 6-month follow-up. The comprehensive CMR assessment included cine imaging for volumetric and feature-tracking strain analysis, T1 and T2 mapping, and late gadolinium enhancement (LGE) for infarct size (IS) and microvascular obstruction (MVO) quantification. The myocardial segments were classified as infarcted, adjacent, or remote based on the LGE distribution. The primary outcome was unfavorable LV functional recovery, defined as failure to achieve an absolute increase of >10 percentage points in the LV ejection fraction (LVEF) or a follow-up LVEF ≤ 50%. **Results**: At the 6-month follow-up, significant reverse remodeling was observed: the LVEF improved from 49.0% [40.7–52.4] to 55.8% [47.9–59.9] (*p* < 0.001), the LV end-systolic volume decreased from 91.8 mL [75.1–113.8] to 80.8 mL [61.9–108.3] (*p* = 0.005), and the relative IS decreased from 30.9% [18.9–45.5] to 19.0% [11.1–31.3] of LV mass (*p* < 0.001). At the follow-up, MVO was no longer detectable in any patient. Unfavorable functional recovery occurred in 17 patients (18.3%). In the multivariable analysis, the infarct zone circumferential strain (CS) was the strongest independent predictor of unfavorable recovery (OR 8.81; 95% CI 1.98–39.28; *p* = 0.004), followed by the relative IS (OR 4.02; 95% CI 1.03–15.73; *p* = 0.045) and a lower infarct zone post-contrast T1 (OR 4.40; 95% CI 1.12–17.36; *p* = 0.034). **Conclusions**: Segmental infarct zone characteristics—particularly circumferential strain, post-contrast T1, and infarct size—provide clinically relevant prognostic information for LV functional recovery after STEMI. The infarct zone CS offers incremental predictive value beyond its structural parameters, reflecting the residual contractile reserve and myocardial viability.

## 1. Introduction

Acute myocardial infarction remains a leading cause of morbidity and mortality worldwide, despite advances in diagnostics and reperfusion therapy [1]. Among survivors, the long-term outcomes are primarily determined by adverse left ventricular (LV) remodeling, which predisposes individuals to progressive heart failure, decreased quality of life, and death [2,3]. Therefore, identifying the patients at risk for incomplete functional recovery is essential to guide post-infarction management [4,5,6]. While numerous studies have identified predictors of adverse clinical outcomes after ST-segment elevation myocardial infarction (STEMI), early imaging-based risk stratification strategies capable of identifying patients at risk of incomplete functional recovery remain incompletely defined.

Cardiovascular magnetic resonance (CMR) imaging provides a comprehensive assessment of myocardial injury through multiple complementary techniques, including late gadolinium enhancement (LGE) for infarct quantification, parametric mapping for tissue characterization, and feature-tracking strain analysis for mechanical function assessment [7]. Together, these modalities provide detailed insights into myocardial injury and recovery potential after STEMI. Prior studies have demonstrated that myocardial strain and tissue characterization can predict LV functional improvement following ST-segment elevation myocardial infarction (STEMI) [8,9]. However, most investigators have focused on global rather than regional parameters. While the LV global longitudinal strain (GLS) remains the most extensively studied strain parameter, emerging data indicate that the LV global circumferential strain (GCS) and segmental strain may better characterize infarcted myocardial mechanics and predict adverse ventricular remodeling [10].

Despite these advances, the relative prognostic value of segmental infarct zone parameters compared with global indices remains incompletely defined. Furthermore, the incremental contribution of infarct zone strain beyond its structural parameters, such as infarct size (IS) and tissue-mapping parameters, has not been systematically evaluated.

The present study aimed to determine whether segmental infarct zone functional and structural CMR parameters provide superior prognostic information for predicting LV functional recovery compared with global indices in patients with a first acute STEMI treated with primary percutaneous coronary intervention (PCI). We hypothesized that the infarct zone circumferential strain would independently predict recovery beyond the IS and other tissue characterization parameters.

## 2. Materials and Methods

### 2.1. Study Design and Population

This single-center, prospective observational study included 112 consecutive patients presenting with a first STEMI. Recruitment was conducted between September 2020 and January 2022 at the Department of Cardiology, Hospital of the Lithuanian University of Health Sciences Kauno klinikos. The diagnosis of STEMI was established according to the Fourth Universal Definition of Myocardial Infarction, based on characteristic ischemic symptoms, ST-segment elevation on electrocardiography (ECG), elevated cardiac troponin I levels (upper reference limit 0.04 µg/L), and angiographic documentation of an acutely occluded coronary artery [11]. All patients underwent reperfusion therapy within 24 h of symptom onset, including a primary PCI in 104 patients and thrombolysis before PCI in 8 patients, and received guideline-directed medical therapy in accordance with the 2020 European Society of Cardiology (ESC) guidelines for the management of acute coronary syndromes [12].

### 2.2. Exclusion Criteria and Patient Flow

Patients were excluded if they had a history of ischemic heart disease (previous myocardial infarction, PCI, or coronary artery bypass grafting), moderate or severe valvular heart disease (including prior valve surgery), prior myocarditis, active malignancy, estimated glomerular filtration rate of 30 mL/min/1.73 m^2^, or pregnancy. Additional exclusion criteria were standard contraindications to CMR—such as incompatible ferromagnetic implants or devices, intracranial aneurysm clips, or severe claustrophobia. Patients with atrial fibrillation at the time of CMR acquisition were also excluded due to the potential impact on image quality and strain analysis.

Clinical data were extracted from electronic medical records and included age, sex, comorbidities, pre-admission medications, cardiovascular risk factors, coronary angiography findings, primary PCI procedural details, and in-hospital complications of STEMI. Anthropometric measurements, including weight and height, were recorded. Body surface area (BSA) was calculated using Du Bois formula: BSA = 0.007184 × weight (kg) × 0.425 × height (cm) × 0.725. Body mass index (BMI) was calculated as weight (kg) divided by height (m^2^).

**Figure 1:**
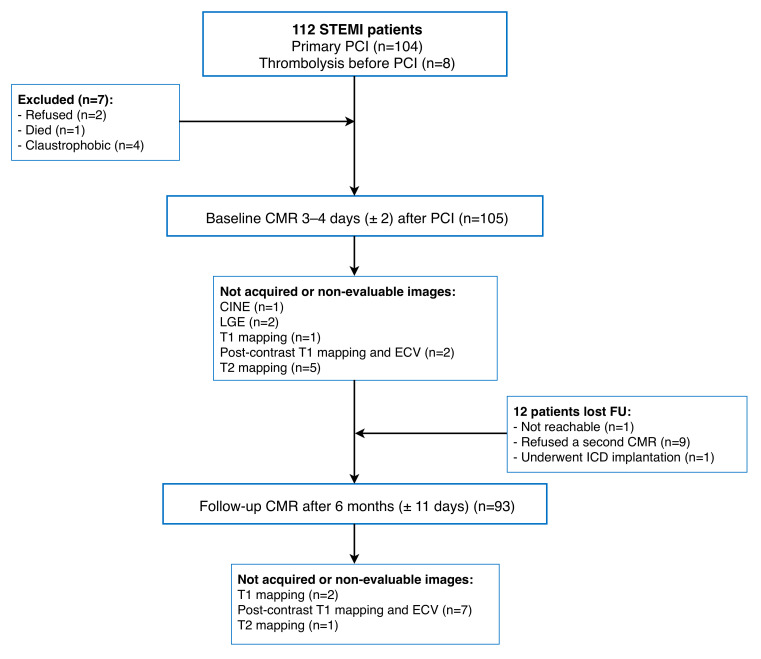
**Study flow diagram.** Of 112 patients with acute STEMI treated with reperfusion therapy, including primary PCI in 104 patients and thrombolysis before PCI in 8 patients, 7 were excluded before baseline imaging and 105 underwent baseline CMR. At 6-month follow-up, 12 patients did not undergo repeat CMR, resulting in 93 patients with paired baseline and follow-up CMR data included in final analysis.

Of the initial 112 patients (Figure 1), 7 were excluded before baseline imaging: 4 due to claustrophobia, 2 declined participation, and 1 died before the baseline scan. Consequently, 105 patients underwent a baseline CMR at a median of 3 days [IQR 2–5] after reperfusion therapy. At the 6-month follow-up, 12 patients did not undergo repeat CMR: 2 were unreachable; 9 declined the follow-up CMR; and 1 had underwent an implantable cardioverter–defibrillator (ICD) implantation, which precluded further CMR imaging. This resulted in 93 patients with paired baseline and follow-up CMR data available for analysis.

The study was conducted in accordance with the Declaration of Helsinki and received approval from the Kaunas Regional Biomedical Research Ethics Committee (Approval No. BE-2-6, approved 5 February 2020). All patients provided written informed consent before enrollment.

### 2.3. CMR Imaging Protocol

All CMR examinations were performed on a 3.0 T clinical scanner (Magnetom Skyra, Siemens Healthineers, Erlangen, Germany) using an 18-channel phased-array body coil in combination with a spine coil. A standardized, comprehensive imaging protocol was applied at both the baseline and follow-up examinations.

The LV cine images were acquired using a balanced steady-state free precession (bSSFP) sequence with retrospective ECG gating during end-expiratory breath-holds. The imaging parameters included: echo time (TE) 1.39 ms; repetition time (TR) 2.5 ms; field of view (FOV) 320–360 mm; matrix 192 × 146; flip angle 47°; slice thickness 8 mm; and inter-slice gap of 2 mm, covering the entire LV from base to apex in contiguous short-axis slices. Standard long-axis views (two, three, and four chamber) were also obtained for comprehensive functional assessment and strain analysis.

Native T1 mapping was performed using an ECG-gated single-shot-modified Look–Locker inversion recovery (MOLLI) sequence with a 5(3)3 acquisition scheme, obtaining eight images over eleven heartbeats with bSSFP readouts. The acquisition parameters were as follows: TE 1.07 ms; TR 2.58 ms; FOV 320–360 mm; matrix 192 × 144; flip angle 35°; slice thickness 8 mm; minimum inversion time 100 ms, with an 80 ms increment; GRAPPA acceleration factor 2; and an imaging window of 136 ms per heartbeat. Post-contrast T1 mapping was performed using the same sequence approximately 15 min after contrast administration.

T2 mapping was performed using a T2-prepared bSSFP sequence with T2 preparation times of 0, 30, and 55 ms, acquiring three images for myocardial T2 quantification and edema assessment.

The reference values for quantitative mapping parameters at our center were established from 25 healthy volunteers (mean age 35 ± 8 years, 52% male) scanned on the same 3.0 T scanner using identical sequences and post-processing protocols: native T1 1250 ± 45 ms, post-contrast T1 385 ± 42 ms, ECV 26.2 ± 2.8%, and T2 43.5 ± 2.5 ms.

The LGE images were obtained 10–15 min following intravenous administration of gadobutrol (0.2 mmol/kg; Gadovist, Bayer Healthcare, Berlin, Germany) using a short-axis stack covering the entire left ventricle. A phase-sensitive inversion-recovery gradient-echo sequence was used, with the inversion time optimized to null the normal myocardial signal according to standard protocols. All acquisitions were performed during end-expiratory breath-holds with ECG gating, and the parameters were chosen based on the Society for Cardiovascular Magnetic Resonance (SCMR) consensus recommendations for standardized acute myocardial infarction assessment [13].

### 2.4. Image Analysis

The CMR images were analyzed offline by a single experienced reader (AV, with >5 years of CMR experience) using commercially available Medis Suite software (version 3.2, Medis Medical Imaging Systems, Leiden, The Netherlands). The reader was blinded to the clinical outcomes and follow-up imaging results at the time of baseline image analysis. The baseline and follow-up CMR datasets were analyzed sequentially and separately, ensuring that post-processing of baseline images was performed without knowledge of follow-up CMR findings. The analysis followed the SCMR standardized guidelines for LV function and mass quantification [14].

The left ventricular end-diastolic (LVEDV) and end-systolic (LVESV) volumes were quantified by tracing the endocardial and epicardial contours on the short-axis cine images at end-diastole and end-systole, respectively, with the papillary muscles and trabeculations included in the LV cavity (blood pool method). The LVEF, left ventricular stroke volume (LVSV), LV myocardial mass, cardiac output (CO), and cardiac index (CI) were derived accordingly. All volumes and myocardial mass values were indexed to the BSA. The volumetric quantification was performed using QMass (Medis Medical Imaging Systems, Leiden, The Netherlands).

A myocardial deformation (strain) analysis was performed using feature tracking with QStrain (Medis Medical Imaging Systems, Leiden, The Netherlands). Long-axis cine views (two chamber, three chamber, and four chamber) were used for global and segmental longitudinal strain assessment, while three short-axis cine images (basal, mid-ventricular, and apical) were used for global and segmental circumferential strain assessment. The endocardial and epicardial contours were defined at end-diastole and automatically propagated through the cardiac cycle using the software’s tissue-tracking algorithm, with manual adjustments applied as needed to ensure accurate tracking. The GLS and GCS were calculated as the average of peak segmental strain values across the American Heart Association (AHA) 16-segment model, with the anterior right ventricular (RV) insertion point used as the reference landmark for segmental assignment [15].

The LGE images were analyzed by manually delineating the endocardial and epicardial contours on all short-axis slices. The IS was quantified using the signal intensity threshold versus reference myocardium (STRM) method, with a threshold of >5 SD above the mean signal intensity of remote myocardium [16,17]. A microvascular obstruction (MVO) was defined as a hypointense core within hyperenhanced regions on the LGE images, and was included in the total IS calculation. The IS and MVO extent were reported both in grams and as a percentage of total LV myocardial mass.

**Figure 2:**
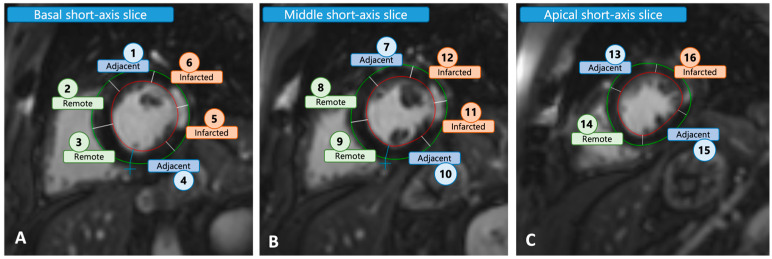
Representative CMR images of myocardial segment classification based on late gadolinium enhancement (LGE) according to the American Heart Association (AHA) 16-segment model. Basal (**A**), mid-ventricular (**B**), and apical (**C**) short-axis slices illustrate infarcted, adjacent, and remote myocardial segments used for segmental strain and parametric mapping analyses. Color-coded labels denote segment classification: orange = infarcted, blue = adjacent, and green = remote myocardium. Numbered labels correspond to the AHA 16-segment model.

The myocardial segments were classified based on the presence of LGE. No minimum LGE extent threshold was applied; any detectable enhancement within a segment, including partially infarcted segments, was sufficient for classification as infarcted. The strain and parametric mapping values were included in the infarcted region averages as whole-segment values, consistent with the segment-level output of the software used. The segments directly bordering the infarcted region were classified as adjacent. All other segments without LGE and not directly adjacent to the infarct zone were defined as remote. This classification was applied consistently in the strain and mapping analyses. A representative CMR example illustrating infarcted, adjacent, and remote myocardial segment classification based on the LGE distribution is shown in Figure 2.

A parametric mapping analysis was performed on three short-axis slices (basal, mid-ventricular, and apical) by tracing the endocardial and epicardial contours, with a 10% offset from the blood pool and epicardial boundaries to minimize partial volume effects. The native and post-contrast T1 and T2 values were measured, and the extracellular volume fraction (ECV) was calculated from the myocardial and blood pool native and post-contrast T1 values, corrected for the patient’s hematocrit obtained within 24 h of CMR examination [18]. For each patient, the native and post-contrast T1, T2, and ECV values were measured in all 16 segments and averaged within the infarcted, adjacent, and remote regions across the three slices. The global mean values were calculated as the weighted average of all segments.

To assess interobserver reproducibility, the CMR feature-tracking strain measurements were re-analyzed in a randomly selected subset of 37 patients by a second independent reader, blinded to the primary reader’s measurements. Agreement was quantified using the intraclass correlation coefficient (ICC, two-way mixed-effects model, absolute agreement definition).

### 2.5. Definition of Outcomes

The primary outcome was unfavorable LV functional recovery at 6 months, defined as failure to achieve an absolute increase in LVEF of >10 percentage points from baseline or a follow-up LVEF ≤ 50%. This composite definition was chosen to capture both patients with inadequate improvement and those with persistently reduced systolic function despite some improvement [19,20]. A secondary outcome was limited myocardial strain recovery, defined as failure to achieve an absolute improvement of >3 percentage points in the LV GLS or GCS at the 6-month follow-up [21,22]. To evaluate the strength of the primary findings, a pre-specified sensitivity analysis was conducted using an alternative outcome definition of impaired LV functional recovery, defined as a follow-up LVEF < 50% irrespective of the magnitude of change from baseline, as previously applied in CMR-based post-STEMI remodeling studies [23].

### 2.6. Statistical Analysis

The continuous variables were expressed as the mean ± standard deviation (SD) for normally distributed data and as the median with interquartile range (IQR) for non-normally distributed data. Normality was assessed using a Shapiro–Wilk test and visual inspection of histograms and Q-Q plots. The categorical variables were presented as counts and percentages.

To compare the normally distributed continuous variables between the baseline and 6-month follow-up, paired Student’s *t*-tests were used. For the non-normally distributed paired data, a Wilcoxon signed-rank test was used. To compare infarcted, adjacent, and remote myocardial segments within the same patient, a one-way repeated-measures analysis of variance (ANOVA) was used for the normally distributed data and a Friedman test was used for the non-normally distributed data. When significant differences among the three myocardial regions were identified, pairwise post hoc comparisons were performed using paired Student’s *t*-tests or Wilcoxon signed-rank tests, as appropriate, with a Bonferroni correction for multiple comparisons. For three pairwise regional comparisons, the adjusted significance threshold was *p* = 0.017.

The categorical variables were compared using a chi-square test or Fisher’s exact test, as appropriate, based on the expected cell frequencies. The correlations between continuous variables were analyzed using Spearman’s rank correlation coefficients, with the correlation strength interpreted as weak (r = 0.10–0.29), moderate (r = 0.30–0.49), or strong (r ≥ 0.50).

Univariable logistic regression analyses were performed to identify the baseline predictors of unfavorable LV functional recovery. Unfavorable LV functional recovery was defined as failure to achieve an absolute increase in LVEF of >10 percentage points from the baseline or a follow-up LVEF ≤ 50%. For the strain parameters, higher, less negative values indicated worse myocardial deformation. The baseline variables, including the LV volumes; LVEF; LV mass; infarct characteristics; strain parameters; and parametric mapping indices across infarcted, adjacent, and remote segments, were first tested in univariable models.

An exploratory multivariable logistic regression analysis was performed using ROC-derived optimal cutoffs for dichotomization of candidate predictors. The cutoff values were selected using a receiver operating characteristic curve analysis. The dichotomized predictors were entered into a forward stepwise Wald logistic regression model. The multivariable model was based on patients with complete data for the selected predictors (*n* = 84). Prior to multivariable entry, the intercorrelations among the candidate predictors were assessed using Spearman’s rank correlation coefficients. The strongly intercorrelated variables were not entered simultaneously: the GCS was excluded in favor of infarcted region CS given their mechanistic overlap, and the LVESV and LVESVi were excluded as mathematical derivatives of infarct extent and global function. Three non-redundant predictors representing distinct dimensions of acute injury were entered: the infarcted region CS (regional mechanics), relative IS (structural burden), and infarcted region post-contrast T1 (tissue integrity). With 17 outcome events and three predictors, the events-per-variable ratio was 5.7; accordingly, the analysis is considered exploratory. The results are reported as odds ratios (ORs) with 95% confidence intervals (CIs).

For the sensitivity analysis, univariable and multivariable logistic regressions were repeated using the alternative outcome definition (follow-up LVEF < 50%). Given the larger number of outcome events, the continuous predictors were entered without dichotomization. Two multivariable models were constructed: a primary model including all significant univariable predictors, and a secondary model excluding the baseline LVEF to evaluate the independent contribution of infarct characteristics and regional strain parameters.

The analytic numbers varied for the individual CMR parameters according to image availability and quality, and are reported in the corresponding tables. All *p*-values are two-sided, with *p* < 0.05 considered statistically significant. No adjustment for multiple comparisons was applied to the exploratory correlation and logistic regression analyses; these findings should therefore be interpreted cautiously. Analyses were conducted using IBM SPSS Statistics version 29.0 (IBM Corp., Armonk, NY, USA).

## 3. Results

### 3.1. Patient Characteristics

A total of 93 patients experiencing a first STEMI underwent CMR imaging at baseline, at a median of 3 days after reperfusion therapy, and at a 6-month follow-up. The median age was 61 years [55–68], and 72 patients (77%) were male. The most prevalent cardiovascular risk factors were dyslipidemia in 85 patients (91%), hypertension in 76 (82%), current smoking in 48 (52%), family history of coronary artery disease in 34 (37%), and diabetes mellitus in 12 (13%). The median BMI was 28.1 kg/m^2^ [25.0–30.8]. Prior to admission, 44 patients (47%) were receiving angiotensin-converting enzyme inhibitors or angiotensin receptor blockers, 30 (32%) were being treated with beta-blockers, and eight (9%) were taking statins.

### 3.2. Procedural Characteristics

The median symptom-to-balloon time was 345 min [198–533], while the median door-to-balloon time was 49 min [33–67]. The culprit vessel was the right coronary artery in 41 patients (44%), the left anterior descending artery in 38 (41%), and the left circumflex artery in 14 (15%). Pre-procedural TIMI 0–1 flow was observed in 67 patients (72%), indicating complete or near-complete coronary occlusion at presentation, while TIMI 3 flow after PCI was achieved in 87 patients (94%). Multivessel disease was present in 45 patients (48%), and non-culprit PCI was performed during the index hospitalization in 22 (24%). Thrombus aspiration was used in 12 patients (13%), and glycoprotein IIb/IIIa inhibitors were administered in four (4%). The median peak troponin concentration was 27.7 µg/L [14.3–49.2]. The baseline clinical and procedural characteristics are summarized in Table 1.

### 3.3. Baseline CMR Findings: Global LV Structure and Function

At baseline (Table 2), the LV volumes were mildly increased, with a median LVEDV of 177.2 mL [149.5–210.0] and LVESV of 91.8 mL [75.1–113.8], corresponding to indexed values of LVEDVi of 90.3 mL/m^2^ [78.4–100.2] and LVESVi of 45.9 mL/m^2^ [37.2–57.3]. The LV mass was within a normal range (114.7 [97.7–132.3] g; LV mass index, 56.2 [49.3–65.3] g/m^2^). The LV systolic function was moderately reduced (LVEF, 49.0% [40.7–52.4]), consistent with the expected degree of dysfunction following a STEMI. The global strain parameters were relatively preserved despite the reduced LVEF, with an LV GLS of −20.4 ± 4.4% and GCS of −27.7 ± 6.7%, suggesting a viable but dysfunctional myocardium with the potential for recovery.

The infarct burden was substantial, with a median IS of 34.7 g [20.7–58.4], corresponding to a relative IS of 30.9% [18.9–45.5] of LV myocardial mass. An MVO was present in 65 patients (69.9%). Among the patients with an MVO, the median MVO mass was 2.8 g [0.0–7.1], corresponding to a relative MVO size of 2.6% [0.0–5.9] of LV mass. An LV thrombus was identified on the baseline CMR in 10 patients (10.8%).

The T1 mapping demonstrated elevated global native T1 (1352.1 ± 70.0 ms), reduced post-contrast T1 (350.1 ± 73.8 ms), and increased ECV (29.5% [26.9–33.1]), reflecting acute myocardial injury and expanded extracellular space. The T2 mapping showed prolonged global T2 values (44.2 ms [42.1–46.4]), consistent with myocardial edema.

### 3.4. Baseline CMR Findings: Segmental LV Structure and Function

At baseline, the LV strain differed across myocardial regions (Table 3). The infarcted segments showed the most severe dysfunction (LS, −17.2% [−20.5 to −13.9]; CS, −25.0% [−30.3 to −19.7]), followed by adjacent segments (LS, −22.0% [−26.7 to −17.6]; CS, −27.2% [−32.3 to −23.7]), while the remote myocardium showed the most preserved circumferential strain values (LS, −21.2% [−25.4 to −17.9]; CS, −30.2% [−36.0 to −25.9]). Pairwise comparisons showed significant differences between the infarcted and remote segments for both LS and CS (*p* < 0.001 for both). The infarcted and adjacent segments differed significantly for LS (*p* < 0.001), whereas the difference in CS did not reach statistical significance (*p* = 0.069). The adjacent and remote segments did not differ significantly for LS (*p* = 1.000), whereas CS differed significantly between the adjacent and remote myocardium (*p* = 0.007).

The tissue characterization parameters followed a similar regional pattern. Compared with the remote myocardium, the infarcted segments demonstrated a significantly higher native T1 (1427.2 ms [1371.9–1479.7] vs. 1262.5 ms [1234.1–1314.0]; *p* < 0.001), higher ECV (36.1% [30.3–41.2] vs. 24.6% [22.5–27.3]; *p* < 0.001), lower post-contrast T1 (321.7 ms [255.2–366.8] vs. 381.5 ms [325.4–434.0]; *p* < 0.001), and higher T2 (47.8 ms [44.3–50.8] vs. 41.3 ms [40.0–43.4]; *p* < 0.001). A similar pattern was observed when comparing the infarcted segments with adjacent myocardium: higher native T1 (1427.2 ms [1371.9–1479.7] vs. 1300.7 ms [1262.0–1345.3]; *p* < 0.001), higher ECV (36.1% [30.3–41.2] vs. 26.2% [23.9–29.0]; *p* < 0.001), lower post-contrast T1 (321.7 ms [255.2–366.8] vs. 374.3 ms [313.1–429.7]; *p* < 0.001), and higher T2 (47.8 ms [44.3–50.8] vs. 42.8 ms [40.4–43.8]; *p* < 0.001).

Adjacent segments also showed evidence of injury compared with the remote myocardium, with higher native T1 (1300.7 ms [1262.0–1345.3] vs. 1262.5 ms [1234.1–1314.0]; *p* < 0.001), higher ECV (26.2% [23.9–29.0] vs. 24.6% [22.5–27.3]; *p* < 0.001), and lower post-contrast T1 (374.3 ms [313.1–429.7] vs. 381.5 ms [325.4–434.0]; *p* = 0.014). The T2 did not differ significantly between the adjacent and remote myocardium (42.8 ms [40.4–43.8] vs. 41.3 ms [40.0–43.4]; *p* = 1.000).

Interobserver reproducibility of feature-tracking strain measurements was assessed in 37 patients. The infarcted region CS demonstrated excellent interobserver agreement (ICC 0.928; 95% CI 0.857–0.963; *p* < 0.001). The infarcted region LS showed good interobserver agreement (ICC 0.804; 95% CI 0.619–0.899; *p* < 0.001). These results further support the superior reliability of circumferential over GLS for infarct zone assessment.

### 3.5. Follow-Up CMR Findings: Global LV Structure and Function

**Figure 3:**
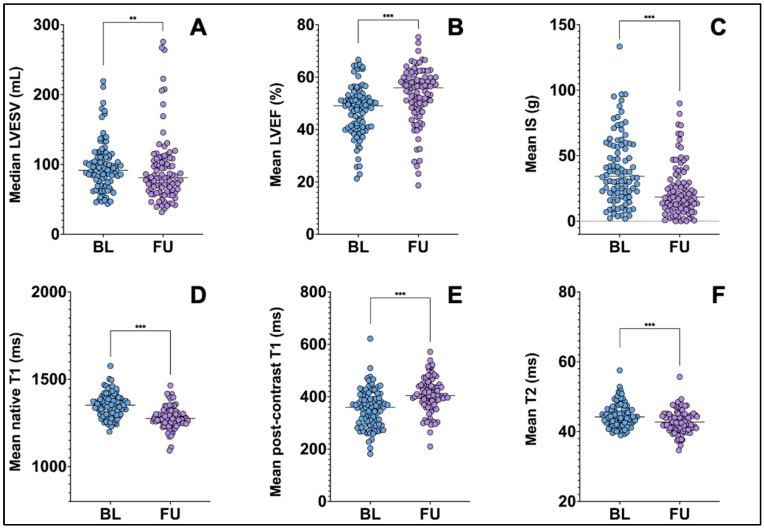
Reverse remodeling and resolution of myocardial injury following STEMI. Scatter plots show individual patient values at baseline (BL) and 6-month follow-up (FU) for (**A**) left ventricular end-systolic volume (LVESV), (**B**) left ventricular ejection fraction (LVEF), (**C**) infarct size, (**D**) native T1, (**E**) post-contrast T1, and (**F**) T2 relaxation time. Each point represents an individual patient; horizontal bars indicate the mean or median, as appropriate. ** *p* < 0.01, *** *p* < 0.001.

At the 6-month follow-up (Table 2), significant reverse remodeling was observed in the majority of patients. The LVEDV was 178.3 mL [146.5–214.5] (*p* = 0.274 vs. baseline), while the LVESV decreased significantly to 80.8 mL [61.9–108.3] (*p* = 0.005) (Figure 3A), with corresponding reductions in the indexed values (LVEDVi 90.2 mL/m^2^ [74.5–103.3], *p* = 0.262; LVESVi 39.9 mL/m^2^ [31.5–51.1], *p* = 0.005). The LVEF improved from 49.0% [40.7–52.4] to 55.8% [47.9–59.9] (*p* < 0.001) (Figure 3B). The LV mass decreased significantly from 114.7 g [97.7–132.3] to 97.2 g [84.8–117.0] (*p* < 0.001), with a corresponding reduction in the LV mass index from 56.2 g/m^2^ [49.3–65.3] to 49.4 g/m^2^ [42.9–54.9] (*p* < 0.001).

The global strain parameters improved significantly, with the LV GLS improving from −20.4 ± 4.4% to −23.5 ± 5.6% (*p* < 0.001) and the GCS improving from −27.7 ± 6.7% to −30.4 ± 8.1% (*p* < 0.001).

The IS decreased substantially, from 34.7 g [20.7–58.4] to 18.5 g [9.8–31.3] (*p* < 0.001) (Figure 3C), corresponding to a reduction in the relative IS from 30.9% [18.9–45.5] to 19.0% [11.1–31.3] of LV mass. An MVO was not detectable at follow-up in any patient.

The global mapping parameters demonstrated resolution of acute injury. The native T1 decreased from 1351.9 ± 69.2 ms to 1281.2 ± 61.3 ms (*p* < 0.001), while the post-contrast T1 increased from 349.2 ± 73.8 ms to 405.6 ± 65.5 ms (*p* < 0.001), consistent with scar maturation and reduced extracellular contrast accumulation (Figure 3D,E). The global ECV remained unchanged, from 29.5% [26.7–33.1] to 28.9% [25.9–32.7] (*p* = 0.334), suggesting limited diffuse interstitial remodeling in this cohort. The global T2 decreased from 44.3 ms [42.1–46.7] to 42.8 ms [41.0–45.1] (*p* < 0.001), reflecting resolution of myocardial edema (Figure 3F).

Scatter plots showing individual patient values at baseline (median 3 days post-PCI) and 6-month follow-up were constructed. Significant reverse remodeling was observed, including a reduction in the left ventricular end-systolic volume (LVESV) (A), an improvement in the left ventricular ejection fraction (LVEF) (B), and a decrease in the IS (C). The tissue characterization demonstrated infarct healing and normalization of myocardial tissue properties, including a decrease in the native T1 (D), an increase in the post-contrast T1 (E), and a reduction in the T2 relaxation time (F), reflecting resolution of myocardial edema. Each point represents an individual patient; the horizontal bars represent the mean or median. Statistical comparisons were performed using paired tests.

### 3.6. Follow-Up CMR Findings: Segmental LV Structure and Function

**Figure 4:**
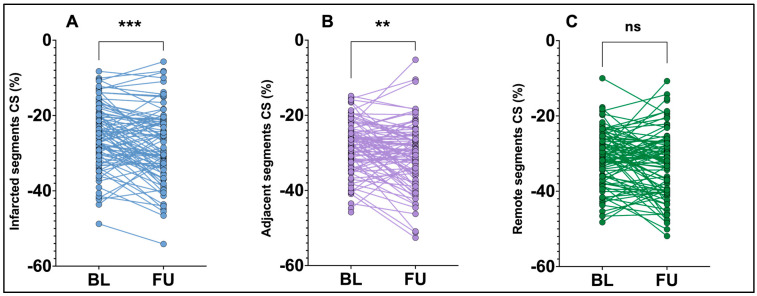
Regional recovery of circumferential myocardial strain following STEMI. Paired plots show individual patient circumferential strain (CS) values at baseline (BL) and 6-month follow-up (FU) in (**A**) infarcted, (**B**) adjacent, and (**C**) remote myocardial segments. Each point represents an individual patient, and connecting lines indicate paired measurements. ** *p* < 0.01, *** *p* < 0.001; ns = not significant.

From the baseline to 6-month follow-up, the strain parameters improved across myocardial regions, with the greatest improvement observed in infarcted segments (Table 4). In infarcted segments, the LS improved from −17.2% [−20.6 to −14.5] to −21.9% [−26.3 to −17.5] (*p* < 0.001), and the CS improved from −25.3% [−30.3 to −19.8] to −29.3% [−33.7 to −21.6] (*p* < 0.001) (Figure 4A). In adjacent segments, the LS did not change significantly, from −21.9% [−26.5 to −17.6] to −23.6% [−29.4 to −19.3] (*p* = 0.091), while the CS improved from −27.2% [−32.2 to −23.7] to −30.1% [−35.7 to −25.7] (*p* = 0.002) (Figure 4B). In remote segments, the LS improved from −21.3% [−25.4 to −18.0] to −25.0% [−28.9 to −21.1] (*p* < 0.001), whereas the CS did not change significantly (−30.2% [−36.0 to −26.0] to −32.3% [−39.7 to −27.8]; *p* = 0.067) (Figure 4C).

The tissue characterization parameters demonstrated resolution of acute injury predominantly in infarcted and adjacent segments. In infarcted segments, the native T1 decreased from 1427.2 ms [1383.0–1481.6] to 1275.0 ms [1248.3–1311.8] (*p* < 0.001), and the post-contrast T1 increased from 317.0 ms [254.8–356.7] to 371.7 ms [331.3–410.6] (*p* < 0.001), while the ECV remained unchanged (*p* = 0.219). In adjacent segments, the native T1 decreased from 1302.1 ms [1259.7–1344.7] to 1262.6 ms [1234.3–1305.3] (*p* < 0.001), the post-contrast T1 increased from 370.5 ms [303.5–429.5] to 423.3 ms [395.8–474.1] (*p* < 0.001), and the ECV remained unchanged (*p* = 0.602). In remote segments, the native T1 showed a modest decrease (1262.7 ms [1235.2–1311.7] to 1253.6 ms [1223.2–1293.4]; *p* = 0.021), the post-contrast T1 increased (375.6 ms [323.0–432.8] to 429.4 ms [397.5–471.2]; *p* < 0.001), and the ECV showed a small but statistically significant increase (24.8% [22.4–27.2] to 25.3% [23.1–27.2]; *p* = 0.002).

The T2 values decreased significantly in infarcted segments (47.8 ms [44.3–50.6] to 43.1 ms [41.5–46.2]; *p* < 0.001), reflecting resolution of edema; did not change significantly in adjacent segments (42.8 ms [40.3–43.9] to 42.1 ms [39.8–44.0]; *p* = 0.396); and showed a modest increase in remote myocardium (41.3 ms [40.0–43.3] to 42.5 ms [39.9–44.4]; *p* = 0.036).

The scatter plots show the individual patients’ circumferential strain (CS) values in infarcted (A), adjacent (B), and remote (C) myocardial segments at baseline (median 3 days post-primary PCI) and 6-month follow-up. The circumferential strain improved significantly in infarcted segments (A) and adjacent segments (B), reflecting recovery of contractile function in previously injured myocardium. No significant change was observed in the remote myocardium (C), consistent with the preserved contractile function at baseline. Each point represents an individual patient; the lines connect paired measurements.

### 3.7. Associations Between Baseline CMR Parameters and 6-Month LV Function

**Figure 5:**
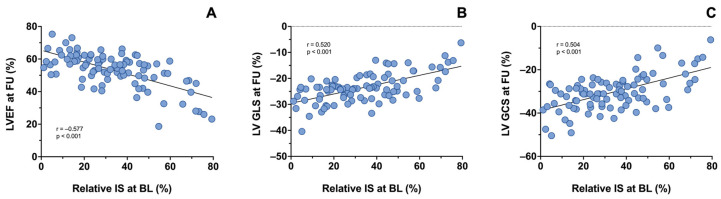
Association between baseline IS and left ventricular functional recovery. Scatter plots showing relationships between baseline IS and left ventricular ejection fraction (LVEF) (**A**), global longitudinal strain (GLS) (**B**), and global circumferential strain (GCS) (**C**) at 6-month follow-up. Larger IS is associated with worse LV functional recovery, reflected by lower LVEF and impaired strain values. Each point represents an individual patient. Solid lines represent linear regression fits. Dotted lines indicate the zero-strain reference (0%).

The baseline IS showed strong associations with the LV functional parameters at the 6-month follow-up (Table 5), including an inverse correlation with the LVEF (r = −0.577; *p* < 0.001) (Figure 5A), indicating that approximately one-third of the variance in the LVEF at follow-up was explained by the baseline IS. Positive correlations were observed with the LV GLS (r = 0.520; *p* < 0.001) and GCS (r = 0.504; *p* < 0.001) (Figure 5B,C), indicating that larger infarcts were associated with worse (less negative) strain values at follow-up. The baseline MVO demonstrated moderate correlations with the LVEF (r = −0.409; *p* < 0.001), LV GLS (r = 0.418; *p* < 0.001), and LV GCS (r = 0.447; *p* < 0.001).

In contrast, the mapping parameters demonstrated weaker associations with functional recovery. The mean native T1 was not significantly associated with the LVEF, LV GLS, or GCS, although its association with the LVEF was borderline (r = −0.187; *p* = 0.073). The mean post-contrast T1 demonstrated a weak correlation with the LVEF (r = 0.233; *p* = 0.025), with no significant associations with the LV GLS or GCS. At the segmental level, the post-contrast T1 in infarcted segments correlated weakly with the LVEF (r = 0.255; *p* = 0.014) and LV GCS (r = −0.223; *p* = 0.033), whereas the association with the LV GLS was not statistically significant (*p* = 0.076). The adjacent native T1 correlated weakly with the LV GLS (r = 0.226; *p* = 0.030), while its associations with the LVEF and LV GCS were not statistically significant. The mean ECV showed a weak inverse correlation with the LVEF (r = −0.235; *p* = 0.023), with no significant associations with the LV GLS or GCS. The segmental ECV in adjacent and remote myocardium was not significantly correlated with the LV functional parameters, while the infarcted ECV showed a weak inverse correlation with the LVEF (r = −0.217; *p* = 0.037) and borderline association with the LV GCS (r = 0.182; *p* = 0.082).

### 3.8. Predictors of Unfavorable LV Functional Recovery

Among the 93 patients with complete baseline and follow-up CMR data, 17 (18.3%) exhibited unfavorable LV functional recovery, defined as failure to achieve an absolute increase in LVEF of >10% from baseline or a follow-up LVEF ≤ 50%. The remaining 76 patients (81.7%) demonstrated favorable recovery.

In the univariable logistic regression analyses (Table 6), unfavorable recovery was associated with a larger relative IS (OR, 1.057 per 1% increase; 95% CI, 1.024–1.091; *p* < 0.001), worse LV GCS (OR, 1.150 per 1% increase; 95% CI, 1.046–1.265; *p* = 0.004), worse infarcted segment circumferential strain (OR, 1.152 per 1% increase; 95% CI, 1.057–1.255; *p* = 0.001), and higher mean ECV (OR, 1.126 per 1% increase; 95% CI, 1.021–1.240; *p* = 0.017). The baseline LVESV and LVESVi were also associated with unfavorable recovery (LVESV: OR, 1.018 per 1 mL increase, 95% CI, 1.004–1.032; *p* = 0.014; LVESVi: OR, 1.038 per 1 mL/m^2^ increase, 95% CI, 1.008–1.069; *p* = 0.013). The LVEDV and LVEDVi showed borderline but non-significant associations with unfavorable recovery (*p* = 0.086 and *p* = 0.058, respectively).

In the exploratory multivariable logistic regression analyses using ROC-derived optimal cutoffs for dichotomization (Table 7), three parameters remained associated with unfavorable LV functional recovery: worse infarcted segment circumferential strain > −16.8% (OR 8.81; 95% CI 1.98–39.28; *p* = 0.004), larger relative IS > 42.3% (OR 4.02; 95% CI 1.03–15.73; *p* = 0.045), and lower infarcted segment post-contrast T1 < 255 ms (OR 4.40; 95% CI 1.12–17.36; *p* = 0.034). Given the limited number of unfavorable recovery events (*n* = 17) and the use of data-driven cutoffs, these multivariable findings should be interpreted with caution and require validation in independent cohorts.

In the pre-specified sensitivity analysis using the alternative outcome definition of follow-up LVEF < 50%, impaired LV functional recovery was observed in 28 patients (30.1%). The patients with a follow-up LVEF < 50% were older; had a lower BMI, were more frequently current smokers; more often presented with anterior MI and pre-PCI TIMI flow 0–1; and had higher peak troponin I, AST, and BNP levels compared with patients with preserved follow-up LVEF (Supplementary Table S1). The baseline LVEF was markedly lower in patients with impaired follow-up LV function (38.5% [33.4–41.8] vs. 50.6% [46.7–55.9]; *p* < 0.001), and the IS was substantially larger (47.2% [33.4–68.1] vs. 26.6% [14.9–38.0]; *p* < 0.001). The infarcted region circumferential strain was also more impaired in this group (−20.1% [−23.7 to −13.5] vs. −27.7% [−32.7 to −23.6]; *p* < 0.001).

In the univariable logistic regression (Supplementary Table S2), impaired follow-up LV function was associated with the baseline LVEF, LVEDVi, LVESVi, GCS, GLS, IS, MVO size, LV thrombus, troponin I, AST, BNP, platelet count, anterior MI, pre-PCI TIMI flow 0–1, current smoking, and infarcted region circumferential strain and ECV, among others.

In the multivariable analysis (Supplementary Table S3), a primary model including the baseline LVEF, troponin I, IS, and infarcted region circumferential strain identified the baseline LVEF as the sole independent predictor of impaired follow-up LV function (OR 0.859; 95% CI 0.758–0.973; *p* = 0.017), with troponin I, IS, and infarcted region CS losing independent significance once the baseline LVEF was accounted for. In a secondary model excluding the baseline LVEF to evaluate the independent contribution of infarct burden and regional mechanics, both a larger IS (OR 1.054; 95% CI 1.015–1.095; *p* = 0.006) and more impaired infarcted region circumferential strain (OR 1.145; 95% CI 1.033–1.269; *p* = 0.010) were independently associated with impaired follow-up LV function.

To formally evaluate the incremental discriminatory value of infarcted region CS beyond structural markers, three sequential logistic regression models were constructed (Supplementary Table S4). Model 1, comprising the relative IS alone, achieved an AUC of 0.772 (95% CI 0.656–0.889). The addition of infarcted region post-contrast T1 in Model 2 did not materially change the discriminatory performance (AUC 0.772; 95% CI 0.655–0.889; ΔLL χ^2^ = 0.503; *p* = 0.478). Further addition of infarcted region CS in Model 3 increased the AUC to 0.800 (95% CI 0.680–0.920), with a ΔAUC of +0.028 and a trend toward improved model fit (ΔLL χ^2^ = 3.033; *p* = 0.082) that did not reach statistical significance, consistent with the limited power imposed by the small event number. All three models demonstrated acceptable calibration (Hosmer–Lemeshow *p* > 0.05 for all).

**Table 7:** Exploratory multivariable logistic regression analysis of ROC-derived baseline CMR predictors of unfavorable LV functional recovery.

Predictor (ROC-Derived Cutoff)	Odds Ratio (95% CI)	*p*-Value
Infarcted segment circumferential strain > −16.8%	8.81 (1.98–39.28)	0.004 *
Relative IS > 42.3%	4.02 (1.03–15.73)	0.045 *
Infarcted segment post-contrast T1 < 255 ms	4.40 (1.12–17.36)	0.034 *

Exploratory multivariable logistic regression is performed using forward stepwise Wald selection with ROC-derived dichotomized predictors. Odds ratios (OR) are presented with 95% confidence intervals (CIs). The analysis includes patients with complete data for the selected predictors. Asterisks (*) indicate statistical significance at *p* < 0.05. Given the limited number of patients with unfavorable LV functional recovery, results should be interpreted with caution. CMR—cardiac magnetic resonance; LV—left ventricular; ROC—receiver operating characteristic.

### 3.9. Clinical Characteristics Associated with Unfavorable Recovery

The patients with unfavorable LV functional recovery differed from those with favorable recovery in several clinical and procedural characteristics (Table 8). Unfavorable recovery was more common in patients with an anterior MI (64.7% vs. 35.5%; *p* = 0.027), those with higher peak troponin levels (61.5 [37.9–118.5] vs. 23.8 [11.2–37.8] µg/L; *p* < 0.001), and those with baseline LV thrombus on CMR (35.3% vs. 5.3%; *p* = 0.002). Multivessel disease was less frequent in the unfavorable recovery group (23.5% vs. 53.9%; *p* = 0.023), and PCI of other arteries during hospitalization was also less frequent in this group (0.0% vs. 28.9%; *p* = 0.010). Age showed a borderline but non-significant difference between groups (67 [57–71] vs. 61 [54–66] years; *p* = 0.059), while symptom-to-balloon time, door-to-balloon time, and most cardiovascular risk factors were comparable between groups.

## 4. Discussion

In this prospective CMR study of patients with a first STEMI treated with PCI, we demonstrate that:

• Segmental infarct zone characteristics provide robust and clinically meaningful prognostic information regarding LV functional recovery.
• While most patients exhibited significant reverse remodeling by 6 months—including improved LVEF, reduced LVESV, complete resolution of MVO, and relative reduction in IS—approximately 18% experienced unfavorable functional recovery.
• The infarct zone circumferential strain, post-contrast T1, and relative IS emerge as the strongest independent predictors of recovery, with CS demonstrating the strongest association and providing incremental prognostic value beyond structural parameters alone.

These findings underscore the central role of infarct zone structural integrity and residual contractile reserve in determining post-infarction functional recovery.

Our results extend prior work by demonstrating that segmental infarct zone functional and tissue characterization parameters outperform global indices at identifying patients at risk of persistent LV dysfunction, supporting the concept that regional myocardial viability, rather than global remodeling metrics alone, determines recovery potential [24,25,26].

### 4.1. Reverse Remodeling and Myocardial Healing After STEMI

The pattern of LV remodeling observed in our cohort is consistent with the established pattern following successful reperfusion. We observed significant reductions in the LVESV, IS, and LV mass, with complete MVO resolution, accompanied by improvements in the LVEF and global strain parameters. The significant reduction in LV mass likely reflects both resolution of myocardial edema and infarct shrinkage, consistent with the observed decreases in the native T1 and T2 values. These findings reflect resolution of myocardial stunning, progressive infarct healing, and adaptive structural remodeling. Podlesnikar et al. similarly demonstrated significant improvements in LV function and strain parameters from 1 week to 6 months post-STEMI, with the LVEF increasing from 48.5 ± 9.5% to 53.5 ± 9.8% (*p* < 0.001), the LVESV decreasing from 71.5 ± 28.5 mL to 62.5 ± 28.5 mL (*p* < 0.001), and the LV GCS improving from −13.8 ± 3.5% to −15.5 ± 3.8% (*p* < 0.001) [27]. Cui et al. reported similar remodeling patterns, with the LVEF improving from 48.9 ± 8.9% at baseline to 54.8 ± 9.1% at 6 months (*p* < 0.001) and the LVESV decreasing from 78.5 ± 31.2 mL to 67.8 ± 29.4 mL (*p* < 0.001) in their cohort [28].

The issue characterization findings paralleled these functional improvements. The native T1 and T2 values decreased significantly within the infarcted segments, reflecting resolution of edema and acute tissue injury, while the post-contrast T1 increased, consistent with scar maturation and reduced extracellular contrast accumulation. These temporal changes align with the known evolution of infarct healing, transitioning from acute inflammation and edema toward organized fibrotic scar formation. Tahir et al. demonstrated that native T1 of infarcted myocardium decreased from 1286 ± 99 ms at baseline (8 ± 5 days) to 1077 ± 50 ms at 6 months (*p* < 0.0001), while T2 decreased from 84 ± 10 ms to 58 ± 4 ms (*p* < 0.0001), with both parameters showing excellent discriminatory ability between acute and chronic infarction (AUC 0.975 and 0.979, respectively) [29]. Hausenloy et al. reported that in the acute phase (4 ± 2 days post-STEMI), the native T1 and T2 values in infarcted myocardium were significantly elevated compared to remote myocardium (T1: 1298 ± 89 ms vs. 1015 ± 42 ms, *p* < 0.001; T2: 68 ± 8 ms vs. 52 ± 4 ms, *p* < 0.001), reflecting acute injury [30].

Notably, the adjacent segments demonstrated intermediate tissue characterization values between those of infarcted and remote myocardium at baseline, with elevated native T1 (1300.7 ms [1262.0–1345.3] vs. 1262.5 ms [1234.1–1314.0]; *p* < 0.001) and ECV (26.2% [23.9–29.0] vs. 24.6% [22.5–27.3]; *p* < 0.001), suggesting a penumbra of tissue injury extending beyond the core infarct zone. These adjacent segments showed significant improvement in circumferential strain at follow-up (CS: from −27.2% [−32.2 to −23.7] to −30.1% [−35.7 to −25.7]; *p* = 0.002), supporting the concept of peri-infarct viable myocardium with recovery potential.

Although the mean ECV remained unchanged (*p* = 0.320), the remote myocardium demonstrated a small but statistically significant increase in the ECV (from 24.8% [22.4–27.2] to 25.3% [23.1–27.2]; *p* = 0.002), suggesting subtle diffuse interstitial remodeling. However, the magnitude of this change was modest (0.5 percentage points) and unlikely to be clinically significant, consistent with the relatively low-risk profile of this cohort treated with contemporary reperfusion and guideline-directed medical therapy. Hausenloy et al. found that while the ECV was significantly elevated in infarcted myocardium (48.5 ± 11.2%) compared to remote myocardium (27.8 ± 3.5%, *p* < 0.001), it could differentiate between salvaged and infarcted tissue, with salvaged myocardium showing intermediate ECV values (32.4 ± 6.8%) [30]. Prior studies have similarly demonstrated that adverse diffuse remodeling may be attenuated in patients with successful reperfusion and preserved remote myocardial integrity [30,31].

### 4.2. Prognostic Importance of Infarct Zone Functional Integrity

Our findings highlight the infarct zone CS as a particularly powerful predictor of LV recovery, outperforming the LS in this context. This difference is mechanistically grounded in the distinct transmural distribution of myocardial fiber populations. The LS is predominantly generated by subendocardial helical fibers, which are highly sensitive to any ischemic injury but lack specificity for transmural injury severity—even modest subendocardial ischemia substantially impairs the GLS, limiting its ability to discriminate between varying degrees of infarct zone damage [22]. The CS, by contrast, primarily reflects the mid-wall circumferentially oriented fibers spanning the full wall thickness, recruited progressively as the ischemic wavefront extends transmurally, making the segmental CS a more specific marker of irreversible injury depth and residual contractile reserve [10,22]. Additionally, the CS is relatively less susceptible to loading conditions and passive tethering from adjacent segments, which may artificially preserve the apparent longitudinal deformation in the peri-infarct zone [20,24]. These properties are consistent with prior work demonstrating the superior discriminatory performance of CS for segmental recovery after STEMI [25].

In our cohort, the infarcted segments demonstrated significant improvement in both the LS and CS (*p* < 0.001 for both), though the strain values remained impaired compared with the remote myocardium at follow-up. This pattern of partial but incomplete recovery supports the concept that infarct zone strain reflects both irreversible injury and recruitable contractile reserve.

Importantly, the infarct zone CS showed the strongest association with unfavorable LV functional recovery in the exploratory multivariable analysis. This underscores its mechanistic relevance beyond simple infarct extent. These findings are consistent with prior studies demonstrating that regional strain abnormalities are closely linked to myocardial viability and subsequent remodeling. Mangion et al. demonstrated that the circumferential strain provides incremental predictive value over infarct extent for segmental functional recovery after STEMI, with each 1% increase in peak circumferential strain associated with a 5–8% increase in odds of segmental improvement [25]. Similarly, Podlesnikar et al. showed that both the LV GCS and GLS strain at 1 week independently predict LVEF normalization at 6 months, with each 1% increase in strain associated with an approximately 41% higher likelihood of LVEF normalization (*p* < 0.001 for both) [27]. Cui et al. identified the infarct zone displacement parameters as independent predictors of reverse LV remodeling (*p* = 0.026) [28].

Our results further reinforce the concept that a segmental functional assessment provides incremental prognostic value beyond global functional measures, reflecting the regional heterogeneity of myocardial injury and recovery potential that may be obscured by global averaging [24,25]. Erley et al. similarly found that global strain values improved within six months after STEMI (GLS from −14 ± 4% to −16 ± 4%, *p* < 0.001; GCS from −15 ± 4% to −16 ± 4%, *p* = 0.023), but the regional strain remained persistently impaired in LGE-positive segments, with the circumferential strain best distinguishing between LGE-negative and -positive segments (AUC 0.73–0.77) [24]. Our findings mirror this pattern, with the infarcted segment strain improving but remaining significantly impaired compared with the remote myocardium at the 6-month follow-up.

A broader contextualization of these findings within the existing literature further supports the primacy of circumferential over LS for infarct zone recovery prediction. Giusca et al., in a study of 74 STEMI patients undergoing serial CMR, demonstrated that the global CS achieved an AUC of 0.86 for identifying preserved follow-up LVEF ≥50%, outperforming the GLS (ΔAUC 0.13; *p* = 0.01) and performing comparably to the LGE-based IS [32]. Muser et al. similarly confirmed, in a cohort of 80 patients with external validation in 222 STEMI cases, that the circumferential strain was the strongest CMR feature-tracking predictor of segmental functional recovery, with its addition to the LGE increasing the overall discriminatory accuracy from 69% to 74%—a benefit particularly pronounced in segments with intermediate LGE transmurality (50–74%), where the CS improved the AUC from 0.60 to 0.75 [33]. Taken together, these data establish a consistent pattern across multiple independent cohorts: the segmental and GCS captures aspects of infarct zone contractile reserve not fully reflected by structural markers, and provides incremental prognostic value particularly in mechanically ambiguous myocardial regions [34]. By contrast, Reindl et al., in the largest CMR-FT cohort to date (*n* = 232), found that the GLS but not GCS was an independent predictor of adverse remodeling defined by volume expansion, suggesting that the relative prognostic superiority of CS vs. GLS may depend on the specific endpoint examined—functional recovery versus volumetric remodeling—and underlines the importance of outcome definition in comparative strain studies, as reflected in the primary and sensitivity analyses of the present study [35].

### 4.3. Tissue Characterization and Infarct Structural Integrity

The post-contrast T1 mapping within infarcted segments also predicted recovery in our cohort. Lower post-contrast T1 values reflected increased gadolinium accumulation, corresponding to expanded extracellular space due to necrosis and fibrosis [36]. This finding supports prior work demonstrating that parametric mapping provides a quantitative assessment of myocardial structural damage and complements IS measurements derived from LGE [31].

Although selected mapping parameters, particularly ECV-related indices, showed associations with unfavorable recovery in the univariable analyses, their independent predictive value was attenuated when the IS and strain parameters were included. This suggests that these mapping parameters primarily reflect infarct severity rather than providing independent prognostic information beyond infarct extent and mechanical function. Chen et al. similarly found that contrast-enhanced parameters (ECV and IS by LGE) were superior to native T1 and T2 mapping for predicting adverse LV remodeling at 6 months, with contrast-enhanced parameters demonstrating better discriminative performance. In their multivariable analysis, only the LGE-based IS and ECV remained independent predictors of adverse remodeling [31].

Our findings align with previous studies demonstrating that the IS remains the dominant structural determinant of LV remodeling and outcome after STEMI, remaining associated with unfavorable recovery in exploratory multivariable analyses [31,37]. Wang et al. found that the IS was the strongest predictor of LVEF recovery, with patients who showed LVEF improvement having a significantly smaller IS [37]. The MVO, while associated with worse outcomes in the acute phase and present in 65 patients (69.9%) at baseline, were completely resolved at follow-up and did not independently predict recovery after adjustment for the IS and myocardial strain. However, Cui et al. found that the MVO mass was an independent predictor of reverse remodeling (*p* = 0.041), suggesting that the extent of microvascular injury may influence the recovery course in some populations [28]. The complete resolution of MVO in our cohort likely reflects its role as an early marker of injury severity rather than a direct determinant of long-term function, consistent with recent evidence that MVO typically resolves within weeks after STEMI and that persistent MVO at follow-up, rather than acute MVO alone, carries the strongest prognostic implications. Lechner et al. demonstrated that persistent MVO at 6-month follow-up was associated with significantly worse outcomes compared to resolved MVO, with a hazard ratio of 2.8 (*p* = 0.001) for major adverse cardiovascular events [38].

### 4.4. Integration of Structural and Functional Parameters

Our findings support a multiparametric model of post-infarction recovery that integrates the structural and functional markers of myocardial injury. The IS reflects the extent of irreversible myocardial damage, the post-contrast T1 characterizes tissue structural integrity, and the circumferential strain reflects residual contractile function. Together, these parameters capture complementary aspects of myocardial injury and recovery potential, with the strain providing information about viable but dysfunctional myocardium that may not be fully captured by tissue characterization alone.

In our exploratory multivariable analysis, the infarcted segment CS, relative IS, and infarcted segment post-contrast T1 were evaluated as complementary predictors of unfavorable recovery. While these findings require validation, given the limited number of events (*n* = 17), they support the prognostic relevance of infarct zone functional assessment beyond structural parameters alone.

This integrated approach aligns with emerging evidence that combining tissue characterization with functional assessment improves prognostic accuracy compared with individual parameters alone [24,25,26]. The observed association of infarct zone CS with unfavorable recovery beyond that of structural parameters suggests that a mechanical function assessment captures aspects of myocardial viability—such as contractile reserve and fiber integrity—that are not fully reflected by tissue composition metrics. Mangion et al. demonstrated that combining the circumferential strain with infarct transmurality improved prediction of segmental recovery, with the combined model achieving superior discrimination (AUC 0.82) compared to either parameter alone (AUC 0.74 for strain, 0.76 for transmurality) [25]. Similarly, Chen et al. showed that combining the ECV with IS yielded better predictive performance (AUC 0.82) than either parameter individually for adverse remodeling [31]. Such multiparametric CMR strategies may enable more precise risk stratification and individualized management following STEMI, potentially identifying patients who would benefit from intensified medical therapy or closer surveillance.

The clinical applicability of infarct zone CS warrants consideration. The feature-tracking analysis was performed post hoc on standard cine sequences without additional acquisition requirements, and the infarcted region CS demonstrated excellent interobserver reproducibility in our study (ICC 0.928; 95% CI 0.857–0.963). However, the feature-tracking strain values showed moderate vendor dependency, and measurements derived from Medis QStrain may not be directly comparable to those from other software platforms. The vendor-specific reference ranges and multivendor validation of the prognostic cutoff identified here (infarcted CS > −16.8%) are required before broader clinical implementation.

The IS and baseline LVEF remain the most accessible markers for post-STEMI risk stratification, and our data confirm their strong associations with functional recovery. The infarct zone CS should be viewed as a complementary parameter rather than a replacement, potentially refining risk stratification in patients with intermediate IS where structural markers alone provide insufficient prognostic certainty. The sequential model analysis demonstrates that IS alone achieves an AUC of 0.772, with the addition of infarcted CS yielding a numerically meaningful but statistically non-significant ΔAUC of +0.028, limited by the current sample size. Until larger prospective studies can confirm its incremental utility, the infarct zone CS is best positioned as a research tool to guide patient selection for intensified surveillance or therapeutic trials.

### 4.5. Limitations

Several limitations warrant consideration. The primary limitation is the small single-center cohort (*n* = 93), which constrains the statistical power and generalizability. Of 112 initially enrolled patients, 19 were excluded due to incomplete paired CMR data—predominantly due to death, clinical deterioration, or withdrawal—potentially introducing survivor bias by preferentially removing higher-risk patients. The cohort represents a relatively low-risk population with successful reperfusion and contemporary medical therapy, limiting the applicability to higher-risk STEMI populations. Prospective multicenter validation is required to establish external validity.

The limited number of outcome events (*n* = 17) constrains the multivariable model robustness, with an events-per-variable ratio of 5.7, which is below the recommended threshold of 10. The wide confidence intervals reflect this instability, and formal discrimination and calibration metrics were not computed given their limited reliability with small event numbers. Penalized regression or bootstrap validation are recommended for future studies.

Additional methodological limitations include: classification of partially infarcted segments based on any detectable LGE without a transmurality threshold, with whole-segment strain and mapping values included in the infarcted region averages; baseline CMR timing variability within a narrow window (range 2–4 days) that may have modestly influenced edema-sensitive parameters, such as native T1 and T2; and the absence of follow-up medication data, precluding formal comparison of neurohormonal therapy between outcome groups, though all patients received guideline-directed therapy at discharge. Intraobserver reproducibility was not formally assessed. The 6-month follow-up may not have captured late remodeling or hard clinical endpoints, and the incremental clinical utility of infarct zone CS beyond simpler markers requires validation in larger prospective studies with mortality and heart failure endpoints.

## 5. Conclusions

In patients with a first STEMI treated with reperfusion therapy, the segmental infarct zone characteristics, particularly the circumferential strain, post-contrast T1, and IS, provided clinically relevant prognostic information regarding LV functional recovery. The infarct zone CS showed the strongest association with unfavorable recovery in the exploratory multivariable analysis, supporting its potential incremental value beyond that of structural parameters, and reflecting residual contractile reserve and myocardial viability. These findings support a multiparametric CMR approach integrating structural and functional assessments for risk stratification following STEMI.

## Figures and Tables

**Table 1:** Baseline clinical and procedural characteristics (*n* = 93).

Characteristic	Overall (*n* = 93)
Demographics and cardiovascular risk factors	
Age, years	61 [55–68]
Male sex, *n* (%)	72 (77.4)
BMI, kg/m^2^	28.1 [25.0–30.8]
Family history of CAD, *n* (%)	34 (36.6)
Diabetes, *n* (%)	12 (12.9)
Hypertension, *n* (%)	76 (81.7)
Dyslipidemia, *n* (%)	85 (91.4)
Active smoking, *n* (%)	48 (51.6)
Regular alcohol consumption, *n* (%)	20 (21.5)
Medical treatment on admission	
- ACE inhibitor or ARB, *n* (%)	44 (47.3)
- Beta-blockers, *n* (%)	30 (32.3)
- Statins, *n* (%)	8 (8.6)
- Antiplatelet drugs, *n* (%)	2 (2.2)
Procedural characteristics	
Symptom-to-balloon, min	345 [198–533]
Door-to-balloon, min	49 [33–67]
Thrombolysis before pPCI, *n* (%)	6 (6.5)
Culprit artery—LAD, *n* (%)	38 (40.9)
Culprit artery—RCA, *n* (%)	41 (44.1)
Culprit artery—LCX, *n* (%)	14 (15.1)
Pre-primary PCI TIMI flow 0–1, *n* (%)	67 (72.0)
Post-primary PCI TIMI flow 3, *n* (%)	87 (93.5)
Multivessel disease, *n* (%)	45 (48.4)
PCI of other arteries during hospitalization, *n* (%)	22 (23.7)
Thrombus aspiration, *n* (%)	12 (12.9)
GPIIb/IIIa inhibitor use, *n* (%)	4 (4.3)
Markers of myocardial injury and complications	
Peak troponin, μg/L	27.7 [14.3–49.2]
Killip class ≥ 2, *n* (%)	22 (23.7)
Atrial fibrillation/flutter, *n* (%)	5 (5.4)
Ventricular tachycardia/fibrillation, *n* (%)	8 (8.6)
Second–third-degree AV block, *n* (%)	4 (4.3)

Values are presented as mean ± standard deviation (SD) for normally distributed data, and as median [interquartile range, IQR] for non-normally distributed data. Percentages are calculated per patient. Door-to-balloon time is time from hospital arrival to first device deployment. Symptom-to-balloon time is the time from symptom onset to the first device deployment. BMI = body mass index; CAD = coronary artery disease; ACE = angiotensin-converting enzyme; ARB = angiotensin receptor blocker; LAD = left anterior descending artery; RCA = right coronary artery; LCX = left circumflex artery; TIMI = thrombolysis in myocardial infarction; PCI = percutaneous coronary intervention; GPIIb/IIIa = glycoprotein IIb/IIIa inhibitor; AV = atrioventricular.

**Table 2:** Changes in CMR parameters from baseline to 6-month follow-up in STEMI patients (*n* = 93).

Parameter	Baseline	6-Month FU	*p*-Value
HR, beats/min	68.5 ± 10.4	62.4 ± 10.7	<0.001 *
LVEDV, mL	177.2 [149.5–210.0]	178.3 [146.5–214.5]	0.274
LVEDVi, mL/m^2^	90.3 [78.4–100.2]	90.2 [74.5–103.3]	0.262
LVESV, mL	91.8 [75.1–113.8]	80.8 [61.9–108.3]	0.005 *
LVESVi, mL/m^2^	45.9 [37.2–57.3]	39.9 [31.5–51.1]	0.005 *
LVSV, mL	84.4 ± 20.5	96.2 ± 22.5	<0.001 *
LVSVi, mL/m^2^	42.1 ± 9.4	48.0 ± 10.1	<0.001 *
LV mass, g	114.7 [97.7–132.3]	97.2 [84.8–117.0]	<0.001 *
LV mass index, g/m^2^	56.2 [49.3–65.3]	49.4 [42.9–54.9]	<0.001 *
CO, L/min	5.7 ± 1.3	5.9 ± 1.3	0.106
CI, L/min/m^2^	2.8 ± 0.6	2.9 ± 0.6	0.115
LVEF, %	49.0 [40.7–52.4]	55.8 [47.9–59.9]	<0.001 *
LV GLS, %	−20.4 ± 4.4	−23.5 ± 5.6	<0.001 *
LV GCS, %	−27.7 ± 6.7	−30.4 ± 8.1	<0.001 *
IS, g	34.7 [20.7–58.4]	18.5 [9.8–31.3]	<0.001 *
Relative IS, %	30.9 [18.9–45.5]	19.0 [11.1–31.3]	<0.001 *
MVO size, g	2.8 [0.0–7.1]	Not detectable	—
Relative MVO size, %	2.6 [0.0–5.9]	Not detectable	—
LV thrombus on CMR, *n* (%)	10 (10.8)	2 (2.2)	0.008 *

Values are presented as mean ± standard deviation (SD) for normally distributed data and median [interquartile range, IQR] for non-normally distributed data. Changes from baseline to 6-month follow-up are assessed using paired *t*-tests or Wilcoxon signed-rank tests, as appropriate. Baseline MVO size and relative MVO size are reported for patients with MVO present on baseline CMR (*n* = 65). Asterisks (*) indicate statistical significance at *p* < 0.05. HR = heart rate; LV = left ventricle/left ventricular; LVEDV = left ventricular end-diastolic volume; LVEDVi = left ventricular end-diastolic volume index; LVESV = left ventricular end-systolic volume; LVESVi = left ventricular end-systolic volume index; LVSV = left ventricular stroke volume; LVSVi = left ventricular stroke volume index; CO = cardiac output; CI = cardiac index; LVEF = left ventricular ejection fraction; GLS = global longitudinal strain; GCS = global circumferential strain; IS = infarct size; MVO = microvascular obstruction; CMR = cardiovascular magnetic resonance.

**Table 3:** Baseline segmental myocardial characteristics assessed by CMR in infarcted, adjacent, and remote segments.

Parameter	Infarcted	Adjacent	Remote	*p*-Value (I vs. A)	*p*-Value (I vs. R)	*p*-Value (A vs. R)	*p*-Value (Overall)
Native T1, ms (*n* = 90)	1427.2 [1371.9–1479.7]	1300.7 [1262.0–1345.3]	1262.5 [1234.1–1314.0]	<0.001 *	<0.001 *	<0.001 *	<0.001 *
Post-contrast T1, ms (*n* = 90)	321.7 [255.2–366.8]	374.3 [313.1–429.7]	381.5 [325.4–434.0]	<0.001 *	<0.001 *	0.014 *	<0.001 *
ECV, % (*n* = 90)	36.1 [30.3–41.2]	26.2 [23.9–29.0]	24.6 [22.5–27.3]	<0.001 *	<0.001 *	<0.001 *	<0.001 *
T2, ms (*n* = 86)	47.8 [44.3–50.8]	42.8 [40.4–43.8]	41.3 [40.0–43.4]	<0.001 *	<0.001 *	1.000	<0.001 *
LS, % (*n* = 89)	−17.2 [−20.5 to −13.9]	−22.0 [−26.7 to −17.6]	−21.2 [−25.4 to −17.9]	<0.001 *	<0.001 *	1.000	<0.001 *
CS, % (*n* = 90)	−25.0 [−30.3 to −19.7]	−27.2 [−32.3 to −23.7]	−30.2 [−36.0 to −25.9]	0.069	<0.001 *	0.007 *	<0.001 *

Analysis is based on 93 patients with paired CMR data; due to image quality or acquisition issues, analytic number for individual parameters ranged from 86 to 90, as indicated in parentheses next to each parameter. Segments are classified as infarcted (I), adjacent (A), or remote (R) according to LGE, as described in Methods Section. Values are presented as median [IQR]. Overall comparisons are performed using Friedman test, with post hoc Wilcoxon signed-rank tests and Bonferroni correction. Asterisks (*) indicate statistical significance at *p* < 0.05. I = infarcted; A = adjacent; R = remote; CMR = cardiovascular magnetic resonance; ECV = extracellular volume; LS = longitudinal strain; CS = circumferential strain; T1 = T1 relaxation time; T2 = T2 relaxation time.

**Table 4:** Segmental myocardial tissue characteristics and strain at baseline and 6-month follow-up.

Segment and Parameter	Baseline	6-Month Follow-Up	*p*-Value
Infarcted segments			
- LS, % (*n* = 91)	−17.2 [−20.6 to −14.5]	−21.9 [−26.3 to −17.5]	<0.001 *
- CS, % (*n* = 91)	−25.3 [−30.3 to −19.8]	−29.3 [−33.7 to −21.6]	<0.001 *
- Native T1, ms (*n* = 91)	1427.2 [1383.0–1481.6]	1275.0 [1248.3–1311.8]	<0.001 *
- Post-contrast T1, ms (*n* = 86)	317.0 [254.8–356.7]	371.7 [331.3–410.6]	<0.001 *
- ECV, % (*n* = 86)	35.9 [30.3–41.3]	34.3 [29.5–41.3]	0.219
- T2, ms (*n* = 89)	47.8 [44.3–50.6]	43.1 [41.5–46.2]	<0.001 *
Adjacent segments			
- LS, % (*n* = 90)	−21.9 [−26.5 to −17.6]	−23.6 [−29.4 to −19.3]	0.091
- CS, % (*n* = 91)	−27.2 [−32.2 to −23.7]	−30.1 [−35.7 to −25.7]	0.002 *
- Native T1, ms (*n* = 91)	1302.1 [1259.7–1344.7]	1262.6 [1234.3–1305.3]	<0.001 *
- Post-contrast T1, ms (*n* = 86)	370.5 [303.5–429.5]	423.3 [395.8–474.1]	<0.001 *
- ECV, % (*n* = 86)	26.1 [23.8–28.8]	26.0 [23.7–28.8]	0.602
- T2, ms (*n* = 89)	42.8 [40.3–43.9]	42.1 [39.8–44.0]	0.396
Remote segments			
- LS, % (*n* = 89)	−21.3 [−25.4 to −18.0]	−25.0 [−28.9 to −21.1]	<0.001 *
- CS, % (*n* = 89)	−30.2 [−36.0 to −26.0]	−32.3 [−39.7 to −27.8]	0.067
- Native T1, ms (*n* = 89)	1262.7 [1235.2–1311.7]	1253.6 [1223.2–1293.4]	0.021 *
- Post-contrast T1, ms (*n* = 84)	375.6 [323.0–432.8]	429.4 [397.5–471.2]	<0.001 *
- ECV, % (*n* = 84)	24.8 [22.4–27.2]	25.3 [23.1–27.2]	0.002 *
- T2, ms (*n* = 87)	41.3 [40.0–43.3]	42.5 [39.9–44.4]	0.036 *

Values are presented as median [IQR]. Analytic number for each paired comparison is shown in parentheses next to each parameter. Paired comparisons between baseline and 6-month follow-up are performed using Wilcoxon signed-rank tests. Asterisks (*) indicate statistical significance at *p* < 0.05. LS = longitudinal strain; CS = circumferential strain; T1 = T1 relaxation time; ECV = extracellular volume; T2 = T2 relaxation time.

**Table 5:** Correlations between baseline CMR structural parameters and left ventricular function at 6-month follow-up.

Predictor	LVEF (r)	LVEF (*p*)	LV GLS (r)	LV GLS (*p*)	LV GCS (r)	LV GCS (*p*)
Structural parameters						
IS, g	−0.573	<0.001 *	0.544	<0.001 *	0.503	<0.001 *
Relative IS, %	−0.577	<0.001 *	0.520	<0.001 *	0.504	<0.001 *
MVO, g	−0.409	<0.001 *	0.418	<0.001 *	0.447	<0.001 *
Relative MVO, %	−0.391	0.001 *	0.395	0.001 *	0.431	<0.001 *
Parametric mapping						
Infarcted T1 post, ms	0.255	0.014 *	−0.186	0.076 †	−0.223	0.033 *
Infarcted ECV, %	−0.217	0.037 *	0.106	0.316	0.182	0.082 †
Adjacent T1 (N), ms	−0.098	0.350	0.226	0.030 *	0.182	0.083 †
Adjacent ECV, %	−0.044	0.673	−0.038	0.720	0.019	0.857
Remote ECV, %	−0.082	0.441	0.050	0.645	0.029	0.788
Mean T1 (N), ms	−0.187	0.073 †	0.121	0.252	0.138	0.189
Mean T1 post, ms	0.233	0.025 *	−0.165	0.117	−0.186	0.076 †
Mean ECV, %	−0.235	0.023 *	0.070	0.508	0.155	0.141

Values are presented as Spearman’s correlation coefficients (r) with corresponding *p*-values. Spearman’s rank correlation is used due to non-normal distributions of several parameters. MVO correlation analyses are performed only in patients with MVO present on baseline CMR (*n* = 65). Asterisks (*) indicate statistical significance at *p* < 0.05; daggers (†) indicate borderline significance (0.05 ≤ *p* < 0.10). These correlation analyses are exploratory, and *p*-values are not adjusted for multiple comparisons. LV = left ventricle; EF = ejection fraction; GLS = global longitudinal strain; GCS = global circumferential strain; MVO = microvascular obstruction; T1 (N) = native T1 relaxation time; T1 post = post-contrast T1 relaxation time; ECV = extracellular volume fraction; CMR = cardiac magnetic resonance.

**Table 6:** Predictors of unfavorable LVEF outcome (*n* = 93).

Baseline Predictor	Univariable OR (95% CI)	*p*-Value
GLS, %	1.095 (0.967–1.239)	0.154
GCS, %	1.150 (1.046–1.265)	0.004 *
LVEDV, mL	1.011 (0.999–1.023)	0.086
LVEDVi, mL/m^2^	1.025 (0.999–1.051)	0.058 †
LVESV, mL	1.018 (1.004–1.032)	0.014 *
LVESVi, mL/m^2^	1.038 (1.008–1.069)	0.013 *
LV mass index, g/m^2^	1.002 (0.960–1.047)	0.914
Relative IS, %	1.057 (1.024–1.091)	<0.001 *
Mean T1N, ms	1.002 (0.994–1.009)	0.688
Mean post-contrast T1, ms	0.994 (0.986–1.002)	0.121
Mean ECV, %	1.126 (1.021–1.240)	0.017 *
Mean T2, ms	1.027 (0.882–1.196)	0.729
Infarcted LS, %	1.067 (0.954–1.193)	0.254
Infarcted CS, %	1.152 (1.057–1.255)	0.001 *
Infarcted T1 N, ms	1.000 (0.994–1.007)	0.944
Infarcted post-contrast T1, ms	0.993 (0.984–1.001)	0.075
Infarcted ECV, %	1.103 (1.031–1.181)	0.005 *
Infarcted T2, ms	1.055 (0.944–1.179)	0.347
Adjacent LS, %	1.051 (0.969–1.141)	0.229
Adjacent CS, %	1.087 (0.995–1.188)	0.065
Adjacent T1 N, ms	1.003 (0.995–1.011)	0.472
Adjacent post-contrast T1, ms	0.995 (0.988–1.002)	0.192
Adjacent ECV, %	1.077 (0.934–1.243)	0.306
Adjacent T2, ms	1.031 (0.868–1.224)	0.731
Remote LS, %	1.015 (0.927–1.111)	0.744
Remote CS, %	1.059 (0.982–1.143)	0.138
Remote T1 N, ms	1.002 (0.995–1.008)	0.637
Remote post-contrast T1, ms	0.995 (0.988–1.002)	0.166

Univariable logistic regression analysis of baseline predictors of unfavorable LV functional recovery. Odds ratios (OR) are presented with 95% confidence intervals (CIs) and represent the change in odds per 1-unit increase in the predictor. MVO analyses include patients with baseline MVO only (*n* = 65). The analytic number varies according to data availability for individual predictors. Asterisks (*) indicate statistical significance at *p* < 0.05; daggers (†) indicate borderline significance (0.05 ≤ *p* < 0.10). LV = left ventricle/left ventricular; LVEF = left ventricular ejection fraction; GLS = global longitudinal strain; GCS = global circumferential strain; LS = longitudinal strain; CS = circumferential strain; LVEDV = left ventricular end-diastolic volume; LVEDVi = left ventricular end-diastolic volume index; LVESV = left ventricular end-systolic volume; LVESVi = left ventricular end-systolic volume index; MVO = microvascular obstruction; ECV = extracellular volume fraction; T1 N = native T1 relaxation time; T2 = T2 relaxation time; OR = odds ratio; CI = confidence interval.

**Table 8:** Baseline clinical, procedural, and in-hospital characteristics according to LV functional outcome (*n* = 93).

Characteristic	Favorable Outcome (*n* = 76)	Unfavorable Outcome (*n* = 17)	*p*-Value
Demographics and cardiovascular risk factors			
Age, years	61 [54–66]	67 [57–71]	0.059
Male sex, *n* (%)	58 (76.3)	14 (82.4)	0.754
BMI, kg/m^2^	28.2 [25.1–30.9]	27.7 [24.0–30.2]	0.612
Family history of CAD, *n* (%)	26 (34.2)	8 (47.1)	0.320
Diabetes, *n* (%)	11 (14.5)	1 (5.9)	0.688
Hypertension, *n* (%)	62 (81.6)	14 (82.4)	1.000
Dyslipidemia, *n* (%)	71 (93.4)	14 (82.4)	0.158
Active smoking, *n* (%)	37 (48.7)	11 (64.7)	0.232
Regular alcohol consumption, *n* (%)	18 (23.7)	2 (11.8)	0.348
Medical treatment on admission			
- ACE inhibitor or ARB, *n* (%)	35 (46.1)	9 (52.9)	0.607
- Beta-blockers, *n* (%)	24 (31.6)	6 (35.3)	0.767
- Statins, *n* (%)	8 (10.5)	0 (0.0)	0.343
- Antiplatelet drugs, *n* (%)	2 (2.6)	0 (0.0)	1.000
Procedural characteristics			
Door-to-balloon time, min	48 [32–63]	66 [34–79]	0.239
Symptom-to-balloon time, min	288 [170–536]	415 [273–565]	0.178
Thrombolysis before pPCI, *n* (%)	4 (5.3)	2 (11.8)	0.301
Anterior MI, *n* (%)	27 (35.5)	11 (64.7)	0.027 *
Pre-primary PCI TIMI flow 0–1, *n* (%)	52 (68.4)	15 (88.2)	0.138
Multivessel disease, *n* (%)	41 (53.9)	4 (23.5)	0.023 *
PCI of other arteries during hospitalization, *n* (%)	22 (28.9)	0 (0.0)	0.010 *
Thrombus aspiration, *n* (%)	8 (10.5)	4 (23.5)	0.222
GPIIb/IIIa inhibitor use, *n* (%)	4 (5.3)	0 (0.0)	1.000
Markers of myocardial injury and complications			
Peak troponin, μg/L	23.8 [11.2–37.8]	61.5 [37.9–118.5]	<0.001 *
Killip class ≥ 2, *n* (%)	17 (22.4)	5 (29.4)	0.539
LV thrombus on baseline CMR, *n* (%)	4 (5.3)	6 (35.3)	0.002 *
Atrial fibrillation/flutter, *n* (%)	4 (5.3)	1 (5.9)	1.000
Ventricular tachycardia/fibrillation, *n* (%)	7 (9.2)	1 (5.9)	1.000
Second–third-degree AV block, *n* (%)	4 (5.3)	0 (0.0)	1.000

Values are presented as median [interquartile range, IQR] or *n* (%), as appropriate. Comparisons between patients with favorable and unfavorable LV functional recovery are performed using Mann–Whitney U test for continuous variables and χ^2^ test or Fisher’s exact test for categorical variables, as appropriate. Asterisks (*) indicate statistical significance at *p* < 0.05. ACE = angiotensin-converting enzyme; ARB = angiotensin receptor blocker; BMI = body mass index; CAD = coronary artery disease; CMR = cardiovascular magnetic resonance; LV = left ventricular; MI = myocardial infarction; PCI = percutaneous coronary intervention; pPCI = primary percutaneous coronary intervention; TIMI = thrombolysis in myocardial infarction; GPIIb/IIIa = glycoprotein IIb/IIIa inhibitor; AV = atrioventricular.

## Data Availability

The data presented in this study are available on request from the corresponding author due to applicable ethical and privacy restrictions.

## Supplementary Materials

The following supporting information can be downloaded at: https://www.mdpi.com/article/10.3390/diagnostics16131992/s1, Table S1. Baseline clinical, laboratory, and CMR characteristics according to follow-up LV functional outcome (sensitivity analysis). Table S2. Univariable binary logistic regression analysis of predictors of impaired follow-up LV function (sensitivity analysis). Table S3. Multivariable binary logistic regression analyses of predictors of impaired follow-up LV function (sensitivity analysis). Table S4. Sequential logistic regression models for incremental predictive value of infarcted region circumferential strain beyond structural parameters.

## Abbreviations

BMI	body mass index
BSA	body surface area
bSSFP	balanced steady-state free precession
CI	cardiac index
CMR	cardiovascular magnetic resonance
CO	cardiac output
CS	circumferential strain
ECG	electrocardiography
ECV	extracellular volume fraction
GCS	global circumferential strain
GLS	global longitudinal strain
IS	infarct size
LGE	late gadolinium enhancement
LS	longitudinal strain
LV	left ventricle/left ventricular
LVEDV	left ventricular end-diastolic volume
LVEDVi	left ventricular end-diastolic volume index
LVEF	left ventricular ejection fraction
LVESV	left ventricular end-systolic volume
LVESVi	left ventricular end-systolic volume index
LVSV	left ventricular stroke volume
LVSVi	left ventricular stroke volume index
MI	myocardial infarction
MVO	microvascular obstruction
PCI	percutaneous coronary intervention
pPCI	primary percutaneous coronary intervention
STEMI	ST-segment elevation myocardial infarction
