# Supplementary material for: Infarct Zone Circumferential Strain Independently Predicts Left Ventricular Functional Recovery After ST-Segment Elevation Myocardial Infarction: A Multiparametric CMR Study
Source: Diagnostics (Basel). 2026 Jun 26;16(13):1992. doi: 10.3390/diagnostics16131992 (PMC13360053; doi:10.3390/diagnostics16131992)
Supplement: Supplementary file 1 [file diagnostics-16-01992-s001.zip › diagnostics-4339819-supplementary.pdf]

Supplementary Material

Supplementary Table S1. Baseline clinical, laboratory, and CMR characteristics according to follow-up LV functional outcome (sensitivity analysis).

**Variable**	**Follow-up LVEF ≥50% (n = 65)**	**Follow-up LVEF <50% (n = 28)**	**p-value**
**Demographics and risk factors**			
Age, years	60.5 (54.0–64.5)	65.0 (57.0–69.5)	**0.047***
Male sex, n (%)	48 (73.8)	24 (85.7)	0.209
BMI, kg/m²	28.4 (25.8–30.9)	26.8 (23.6–29.2)	**0.046***
Diabetes mellitus, n (%)	11 (16.9)	1 (3.6)	0.099
Arterial hypertension, n (%)	55 (84.6)	21 (75.0)	0.271
Dyslipidemia, n (%)	62 (95.4)	23 (82.1)	0.051
Current smoking, n (%)	29 (44.6)	19 (67.9)	**0.045***
Family history of ischemic heart disease, n (%)	25 (38.5)	9 (32.1)	0.562
**Clinical and procedural characteristics**			
Pain-to-balloon time, min	310.0 (152.5–545.0)	365.0 (207.5–510.0)	0.612
Door-to-balloon time, min	41.0 (31.5–59.0)	58.0 (44.5–74.0)	**0.043***
Anterior MI, n (%)	22 (33.8)	16 (57.1)	**0.042***
Pre-PCI TIMI flow 0–1, n (%)	42 (64.6)	25 (89.3)	**0.015***
Killip class ≥2, n (%)	14 (21.5)	8 (28.6)	0.464
**Laboratory and biomarker parameters**			
PLT, ×10⁹/L	222.0 (190.0–242.0)	207.5 (167.3–242.8)	0.084
Troponin I, μg/L	21.6 (9.4–35.7)	52.9 (35.4–92.7)	**<0.001***
BNP, ng/L	143.7 (82.4–236.8)	218.3 (134.3–271.8)	**0.037***
AST, IU/L	131.0 (75.5–190.5)	312.0 (182.5–433.0)	**<0.001***
Urea, mmol/L	5.1 (4.2–6.4)	5.8 (4.5–7.6)	0.194
eGFR, mL/min/1.73 m²	96.0 (82.7–102.5)	93.1 (79.4–98.8)	0.215
**Baseline CMR parameters**			
LVEF, %	50.6 (46.7–55.9)	38.5 (33.4–41.8)	**<0.001***
LVEDVi, mL/m²	84.0 (72.1–96.7)	95.9 (90.5–113.7)	**<0.001***

Variable	Follow-up LVEF ≥50% (n = 65)	Follow-up LVEF <50% (n = 28)	p-value
LVESVi, mL/m^2	41.7 (36.3–51.1)	58.1 (52.8–72.9)	**<0.001***
GLS, %	−21.1 (−23.7 to −18.4)	−18.6 (−21.8 to −15.2)	**0.012***
GCS, %	−29.5 (−33.4 to −25.9)	−22.4 (−26.7 to −18.6)	**<0.001***
Infarct size, %	26.6 (14.9–38.0)	47.2 (33.4–68.1)	**<0.001***
MVO size, % *	3.5 (2.0–5.9)	7.1 (3.3–10.3)	**0.006***
Baseline LV thrombus, n (%)	2 (3.1)	8 (28.6)	**0.042***
**Infarcted region CMR parameters**			
Longitudinal strain, %	−17.4 (−20.2 to −15.0)	−15.8 (−20.7 to −12.5)	0.287
Circumferential strain, %	−27.7 (−32.7 to −23.6)	−20.1 (−23.7 to −13.5)	**<0.001***
Native T1, ms	1429.1 (1368.2–1483.6)	1420.3 (1385.2–1472.7)	0.893
Post-contrast T1, ms	327.0 (284.1–381.4)	288.1 (242.6–338.1)	**0.044***
ECV, %	35.0 (30.7–39.2)	40.3 (29.7–47.1)	0.058
T2, ms	47.5 (44.3–49.5)	48.5 (44.1–54.7)	0.259

Values are presented as median (IQR) or n (%), as appropriate. Continuous variables are compared using Mann–Whitney U test; categorical variables are compared using chi-square test or Fisher's exact test. MVO analyses are performed in patients with MVO present on baseline CMR only (n = 65). Asterisks (*) indicate statistical significance at p < 0.05. Impaired follow-up LV function is defined as follow-up LVEF <50%. AST—aspartate aminotransferase; BMI—body mass index; BNP—B-type natriuretic peptide; CMR—cardiac magnetic resonance; ECV—extracellular volume; eGFR—estimated glomerular filtration rate; GCS—global circumferential strain; GLS—global longitudinal strain; LVEDVi—LV end-diastolic volume index; LVEF—left ventricular ejection fraction; LVESVi—LV end-systolic volume index; MI—myocardial infarction; MVO—microvascular obstruction; PCI—percutaneous coronary intervention; PLT—platelet count; TIMI—thrombolysis in myocardial infarction.

Supplementary Table S2. Univariable binary logistic regression analysis of predictors of impaired follow-up LV function (sensitivity analysis).

Variable	OR	95% CI	p-value
**Demographics and risk factors**			
Age, years	1.036	0.985–1.090	0.172
Male sex	2.125	0.644–7.015	0.216
BMI, kg/m²	0.895	0.794–1.008	0.068
Diabetes mellitus	0.182	0.022–1.483	0.111
Arterial hypertension	0.545	0.184–1.621	0.275
Dyslipidemia	0.223	0.049–1.007	0.051
Current smoking	2.621	1.032–6.654	**0.043***
Family history of ischemic heart disease	0.758	0.297–1.935	0.562
**Clinical and procedural characteristics**			
Pain-to-balloon time, min	1.000	0.998–1.002	0.840
Door-to-balloon time, min	1.001	0.993–1.008	0.839
Anterior MI	2.606	1.051–6.460	**0.039***
Pre-PCI TIMI flow 0–1	4.563	1.242–16.762	**0.022***
Killip class ≥2	1.457	0.530–4.004	0.465
**Laboratory and biomarker parameters**			
PLT, ×10⁹/L	0.989	0.979–1.000	**0.041***
Troponin I, μg/L	1.035	1.017–1.053	**<0.001***
BNP, ng/L	1.004	1.000–1.007	**0.033***
AST, IU/L	1.009	1.005–1.013	**<0.001***
Urea, mmol/L	1.248	0.968–1.608	0.087
eGFR, mL/min/1.73 m²	0.987	0.961–1.014	0.351
**Baseline CMR parameters**			
LVEF, %	0.806	0.734–0.885	**<0.001***
LVEDVi, mL/m²	1.047	1.018–1.075	**0.001***
LVESVi, mL/m²	1.120	1.061–1.182	**<0.001***
GLS, %	1.158	1.033–1.298	**0.012***
GCS, %	1.267	1.134–1.416	**<0.001***

Variable	OR	95% CI	p-value
Infarct size, %	1.080	1.043–1.117	**<0.001***
MVO size, %	1.174	1.051–1.311	**0.004***
Baseline LV thrombus	12.600	2.471–64.251	**0.002***
**Infarcted region CMR parameters**			
Longitudinal strain, %	1.047	0.955–1.148	0.327
Circumferential strain, %	1.211	1.108–1.323	**<0.001***
Native T1, ms	1.001	0.995–1.006	0.779
Post-contrast T1, ms	0.993	0.987–1.000	0.058
ECV, %	1.074	1.014–1.138	**0.015***
T2, ms	1.077	0.982–1.182	0.116

Odds ratios (ORs) represent change in odds per 1-unit increase in each continuous predictor. MVO analyses are performed in patients with MVO present on baseline CMR only (n = 65). Asterisks (*) indicate statistical significance at p < 0.05. Impaired follow-up LV function is defined as follow-up LVEF <50%. AST—aspartate aminotransferase; BNP—B-type natriuretic peptide; CI—confidence interval; CMR—cardiac magnetic resonance; ECV—extracellular volume; eGFR—estimated glomerular filtration rate; GCS—global circumferential strain; GLS—global longitudinal strain; LVEDVi—LV end-diastolic volume index; LVEF—left ventricular ejection fraction; LVESVi—LV end-systolic volume index; MI—myocardial infarction; MVO—microvascular obstruction; OR—odds ratio; PCI—percutaneous coronary intervention; PLT—platelet count; TIMI—thrombolysis in myocardial infarction.

Supplementary Table S3. Multivariable binary logistic regression analyses of predictors of impaired follow-up LV function (sensitivity analysis).

Variable	Primary model OR (95% CI)	p-value	Sensitivity model OR (95% CI)	p-value
Baseline LVEF, %	0.859 (0.758–0.973)	**0.017***	—	—
Troponin I, μg/L	1.018 (0.998–1.038)	0.080	—	—
Baseline infarct size, %	1.020 (0.975–1.067)	0.387	1.054 (1.015–1.095)	**0.006***
Infarcted region circumferential strain, %	1.037 (0.911–1.181)	0.583	1.145 (1.033–1.269)	**0.010***

Primary model includes baseline LVEF, troponin I, infarct size, and infarcted region circumferential strain. Sensitivity model excludes baseline LVEF to assess independent contribution of infarct burden and regional mechanics. Odds ratios (ORs) are presented with 95% confidence intervals (CIs). Asterisks (*) indicate statistical significance at $p < 0.05$. Impaired follow-up LV function is defined as follow-up LVEF <50%. CI—confidence interval; LVEF—left ventricular ejection fraction; OR—odds ratio.

Supplementary Table S4. Sequential logistic regression models for incremental predictive value of infarcted region circumferential strain beyond structural parameters.

	**Model 1**<br>**Infarct size**	**Model 2**<br>**Infarct size + T1**	**Model 3**<br>**Infarct size + T1 + CS**
**Model performance**			
AUC (95% CI)	0.772 (0.656–0.889)	0.772 (0.655–0.889)	0.800 (0.680–0.920)
p-value (vs. AUC = 0.5)	**<0.001***	**<0.001***	**<0.001***
ΔAUC vs. prior model	—	−0.001	+0.028
−2 Log likelihood	74.537	74.034	71.001
Nagelkerke R^2	0.227	0.234	0.279
LR χ^2 vs. prior model (df)	—	0.503 (1)	3.033 (1)
LR p-value vs. prior model	—	0.478	0.082
Hosmer–Lemeshow χ^2	7.084	4.728	4.988
Hosmer–Lemeshow p	0.528	0.786	0.759

Abbreviations: AUC—area under the receiver operating characteristic curve; CI—confidence interval; CS—circumferential strain; df—degrees of freedom; LR—likelihood ratio; OR—odds ratio; T1—post-contrast T1 relaxation time.
Model 1: Relative infarct size only. Model 2: Relative infarct size + infarcted region post-contrast T1. Model 3: Relative infarct size + infarcted region post-contrast T1 + infarcted region circumferential strain. All models use forced entry. Odds ratios represent change in odds per 1-unit increase in each continuous predictor. LR χ^2 is computed as difference in −2 Log likelihood between nested models. Asterisks (*) indicate statistical significance at $p < 0.05$.
